# Patient-reported outcomes after lateral unicompartmental and total knee arthroplasty: a propensity score-matched cohort study

**DOI:** 10.1007/s00402-026-06491-1

**Published:** 2026-08-28

**Authors:** Mustafa Hariri, Reza Sorbi, Babak Moradi, Johannes Weishorn, Raphael Trefzer, Tobias Reiner, Tilman Walker, Timo Albert Nees

**Affiliations:** 1 Department of Orthopaedics, University Hospital Heidelberg, Heidelberg, Germany; 2 Department of Orthopedics and Trauma Surgery, University Hospital Schleswig-Holstein, Kiel, Germany; 3 Orthopedic Research Center Kiel University, Kiel, Germany

**Keywords:** Knee replacement, Unicompartmental, Uni, Lateral, TKA, UKA

## Abstract

**Introduction:**

Comparative evidence on patient-reported outcomes (PROs) after lateral unicompartmental knee arthroplasty (UKA) and total knee arthroplasty (TKA) remains limited. We compared PROs between cohorts matched for demographic characteristics and baseline knee function.

**Materials and methods:**

Prospectively collected single-center data were retrospectively analyzed. Lateral UKA was performed for isolated lateral-compartment osteoarthritis and TKA predominantly for multicompartmental disease. One-to-one propensity score matching incorporated age and body mass index, exact matching on sex, and restrictions on age and preoperative Oxford Knee Score (OKS), yielding 31 pairs. Postoperative OKS was the primary outcome. Secondary outcomes included OKS improvement, Forgotten Joint Score (FJS), University of California, Los Angeles (UCLA) activity score, OKS minimal clinically important difference (MCID) achievement, and FJS patient acceptable symptom state (PASS) attainment.

**Results:**

All matching covariates achieved acceptable balance (absolute standardized mean differences < 0.20). Postoperative OKS did not differ significantly (UKA, 38.9 ± 7.0; TKA, 39.8 ± 7.5; mean paired difference [TKA minus UKA], 0.94; 95% confidence interval [CI], − 2.57 to 4.44; *p* = 0.590). No significant differences were detected for OKS improvement (difference, 0.77; 95% CI, − 2.62 to 4.17; *p* = 0.644) or FJS (difference, 0.83; 95% CI, − 12.53 to 14.19; *p* = 0.900). UCLA scores were higher after TKA (difference, 0.93; 95% CI, 0.26 to 1.60; *p* = 0.008). MCID achievement (UKA, 90.3%; TKA, 93.5%; *p* = 1.000) and PASS attainment (UKA, 67.7%; TKA, 77.4%; *p* = 0.774) did not differ significantly.

**Conclusions:**

No significant differences in postoperative OKS, OKS improvement, or FJS were detected, whereas UCLA scores were higher after TKA. Given differing indications and limited sample size, these findings do not establish equivalence or comparative effectiveness between procedures.

## Introduction

Total knee arthroplasty (TKA) remains the most frequently performed reconstructive procedure for advanced knee osteoarthritis (OA) because it is applicable across a broad spectrum of disease patterns and provides reliable pain relief and functional improvement [11, 33]. However, in patients with predominantly unicompartmental degeneration, unicompartmental knee arthroplasty (UKA) has regained attention due to its bone- and ligament-preserving concept and the potential for a more physiological knee function [12, 20]. Contemporary evidence indicates that UKA can yield excellent patient-reported outcomes (PROs) and rapid early recovery, while also emphasizing that appropriate patient selection and surgeon experience are critical determinants of success [4, 29].

While UKA cohorts often demonstrate favorable early function and lower short-term morbidity, registry-based data consistently report a higher revision risk for UKA compared with TKA, particularly in younger patients [7, 34]. In a large registry analysis, the lifetime risk of revision after UKA was approximately twice that of TKA across age groups, underscoring the importance of careful indication and patient counseling [34]. At the same time, more recent national and institutional datasets suggest that UKA outcomes have continued to improve, reflecting advances in indications, implant design, and surgical technique, and that the revision-risk gap relative to TKA may narrow in experienced settings [3, 4, 24].

Within the spectrum of UKA, lateral UKA represents a distinct and relatively rare entity. Lateral compartment–dominant OA is uncommon, often developing secondary to previous lateral meniscectomy or post-traumatic joint degeneration. As a result, lateral UKA is performed far less frequently than medial UKA in routine clinical practice, resulting in limited institutional experience in many centers [25]. Nonetheless, contemporary series have reported favorable clinical outcomes for lateral UKA when performed in appropriately selected patients [1, 30].

However, direct comparisons between UKA and TKA remain challenging because patients selected for UKA often differ substantially from those undergoing TKA. UKA recipients are typically younger, have better preoperative function, and present with more localized disease, all of which may independently influence postoperative outcomes. Consequently, the functional advantages reported for UKA in unmatched cohorts may partly reflect these baseline differences. Comparative studies controlling for relevant demographic and functional variables have generally reported smaller between-group differences in PROs after medial UKA and TKA [8, 27].

Against this background, lateral-specific comparisons using cohorts matched for selected measurable preoperative characteristics remain scarce. A recent compartment-specific study compared lateral UKA and TKA in patients with isolated lateral-compartment OA and anterior knee pain at 24 months [21]. Nevertheless, evidence addressing joint awareness, activity level, clinically relevant outcome thresholds, and outcomes beyond two years remains limited.

The aim of this study was therefore to compare PRO following lateral UKA and TKA in a propensity score–matched cohort balanced for key demographic characteristics and preoperative function. The study was exploratory, and we hypothesized that between-group differences in postoperative PROs would be attenuated after matching.

## Methods

The present study retrospectively analyzed prospectively collected data from a consecutive series of patients who underwent either lateral UKA or TKA at a single institution. Between 2014 and 2020, a total of 143 lateral UKAs were implanted using a fixed-bearing design (Oxford Fixed Bearing, Zimmer Biomet, Warsaw, Indiana, USA). During a partially overlapping period between 2014 and 2017, 177 TKAs were performed using the Attune Knee System (DePuy Synthes, Warsaw, Indiana, USA).

Inclusion criteria comprised a minimum age of 18 years and signed informed consent.

Patients were excluded if postoperative outcome data were incomplete or missing, if follow-up was less than one year, if informed consent for study participation was not provided or if revision surgery had been performed prior to the scheduled follow-up assessment.

Only cases with the demographic, preoperative OKS, and postoperative outcome data required for the planned analysis were included in the matching procedure. Missing values were not imputed. The primary analysis therefore followed a complete-case approach.

Ethical approval was obtained by the institutional review boards of the University of Heidelberg (S-003/2025) and the study was conducted in accordance with the Helsinki Declaration of 1975, as revised in 2013. Informed consent was obtained from all participating patients.

The primary indication for lateral UKA was isolated lateral compartment OA presenting with full-thickness cartilage loss (“bone-on-bone”). In contrast, the indication for TKA was advanced knee OA involving more than one compartment, including bicompartmental or tricompartmental disease patterns. As part of institutional practice, lateral UKA was implanted whenever the anatomical and biological prerequisites were fulfilled, whereas TKA was selected in cases with more extensive joint degeneration.

For lateral UKA, eligibility criteria included functionally intact anterior cruciate ligament as well as medial and lateral collateral ligaments, a correctable valgus deformity, and absence of relevant OA in the medial compartment on varus stress radiographs. Degenerative changes of the patellofemoral joint were not considered a contraindication unless advanced bone loss or deep eburnation was present. Contraindications for UKA included inflammatory arthritis, fixed valgus deformity, previous osteotomy, or a flexion contracture greater than 15°. Patients undergoing TKA did not have to meet these restrictive criteria and were treated according to standard indications for TKA.

All UKA procedures were performed using a minimally invasive lateral parapatellar approach without patellar dislocation. Implant positioning aimed to restore physiological joint alignment and avoid elevation of the joint line. In the initial lateral UKA cohort, all tibial components were cemented, whereas the femoral component could be implanted cementless when the operating surgeon considered the intraoperative bone quality and primary implant stability sufficient. All 31 lateral UKAs included in the matched analysis had cemented tibial and femoral components. TKA procedures were performed using a standard medial parapatellar approach, and all TKA components were cemented. Cemented components were implanted following pulsatile lavage and thorough drying of the bone surfaces, with bone cement manually applied and pressurized.

Perioperative antibiotic prophylaxis was administered routinely, and postoperative rehabilitation protocols were standardized across both groups, allowing immediate full weight-bearing and unrestricted range of motion from the first postoperative day.

All TKA procedures were performed by or under the direct supervision of seven senior surgeons experienced in knee arthroplasty. A senior surgeon was defined as a surgeon performing more than 50 arthroplasty procedures annually. Lateral UKA procedures were performed by or under the direct supervision of six senior surgeons, five of whom also performed TKA procedures. During the study period, the mean institutional volume was 20 lateral UKA procedures per year. Of the 31 TKA procedures included in the matched analysis, 24 (77.4%) were performed using a cruciate-retaining (CR) design and 7 (22.6%) using a posterior-stabilized (PS) design.

Clinical follow-up examinations were routinely scheduled at 1, 3, and 5 years postoperatively. For the present analysis, the latest available postoperative assessment was used for each case rather than a uniform scheduled follow-up time point. Consequently, the analyzed follow-up interval varied between patients. PRO measures included the Oxford Knee Score (OKS), the Forgotten Joint Score (FJS), and the University of California Los Angeles activity scale (UCLA).

At in-person follow-up visits, patients completed the questionnaires independently. For telephone follow-up, the standardized questionnaire items were read verbatim by trained study personnel, who recorded the patients’ responses without interpretation, prompting, or assistance in selecting an answer.

### Statistical analysis

Data were collected and analyzed using SPSS version 29.0 (SPSS Inc., Chicago, IL) and R (Version 4.3.2; R Foundation for Statistical Computing, Vienna, Austria).

To reduce imbalance in selected measured baseline characteristics, a propensity score matching (PSM) approach was applied using R. Propensity scores were estimated using logistic regression with age at surgery and body mass index (BMI) as covariates. Patients were matched in a 1:1 nearest-neighbor procedure without replacement using a caliper width of 0.2 standard deviations of the logit of the propensity score. In addition, exact matching on sex was performed, and a maximum difference of 3 points in the preoperative OKS and 5 years in age was allowed between matched pairs. Only successfully matched pairs were included in the outcome analyses. Matching variables were selected based on their clinical relevance to treatment selection and postoperative PROs and their availability in both cohorts. Age and BMI were included in the propensity score model, while exact matching on sex was used to prevent residual imbalance in this categorical variable. Additional restrictions on age and preoperative OKS were applied to limit within-pair differences in age and baseline knee function between matched pairs.

Covariate balance before and after PSM was assessed using standardized mean differences (SMDs). Absolute SMDs were interpreted continuously, with values below 0.20 considered indicative of acceptable balance for the present matched cohort.

Because this retrospective study included all eligible cases treated during predefined study periods, the final sample size was determined by the number of pairs satisfying the matching criteria. No prospective sample-size calculation was performed. Statistical precision was assessed by reporting mean paired differences, standardized paired effect sizes (Cohen’s dz), and 95% confidence intervals.

Postoperative OKS was designated as the primary outcome. Secondary outcomes were the pre-to-post OKS change, postoperative FJS, postoperative UCLA activity score, achievement of the OKS minimal clinically important difference (MCID), and attainment of the FJS patient acceptable symptom state (PASS). Preoperative UCLA activity scores and FJS were not available in the analytical dataset and could therefore not be included in the PSM procedure or used to calculate pre-to-postoperative changes.

Clinical relevance of postoperative outcomes was assessed using two established patient-reported thresholds. First, achievement of the MCID was evaluated for the OKS defined as an improvement of at least 5 points from pre- to postoperative assessment. Second, the patient acceptable symptom state (PASS) was applied to the FJS, with a cutoff value of 40.63 points according to published recommendations [36]. The proportion of patients meeting the respective threshold was calculated separately for the UKA and TKA groups. Between-group differences in MCID and PASS proportions were analyzed using exact McNemar tests.

Continuous outcomes are presented as mean ± standard deviation. Because the outcome analysis was based on matched UKA–TKA pairs, between-group comparisons were performed using two-sided paired t-tests. The distribution of the within-pair differences was evaluated separately for each continuous endpoint using Q–Q plots, the Shapiro–Wilk test, and examination for influential outliers. No major violations of the normality assumption were identified.

All statistical tests were two-sided, and the significance level was set at *p* < 0.05. Secondary outcomes were considered exploratory, and no adjustment for multiple comparisons was performed.

## Results

**Fig. 1:**
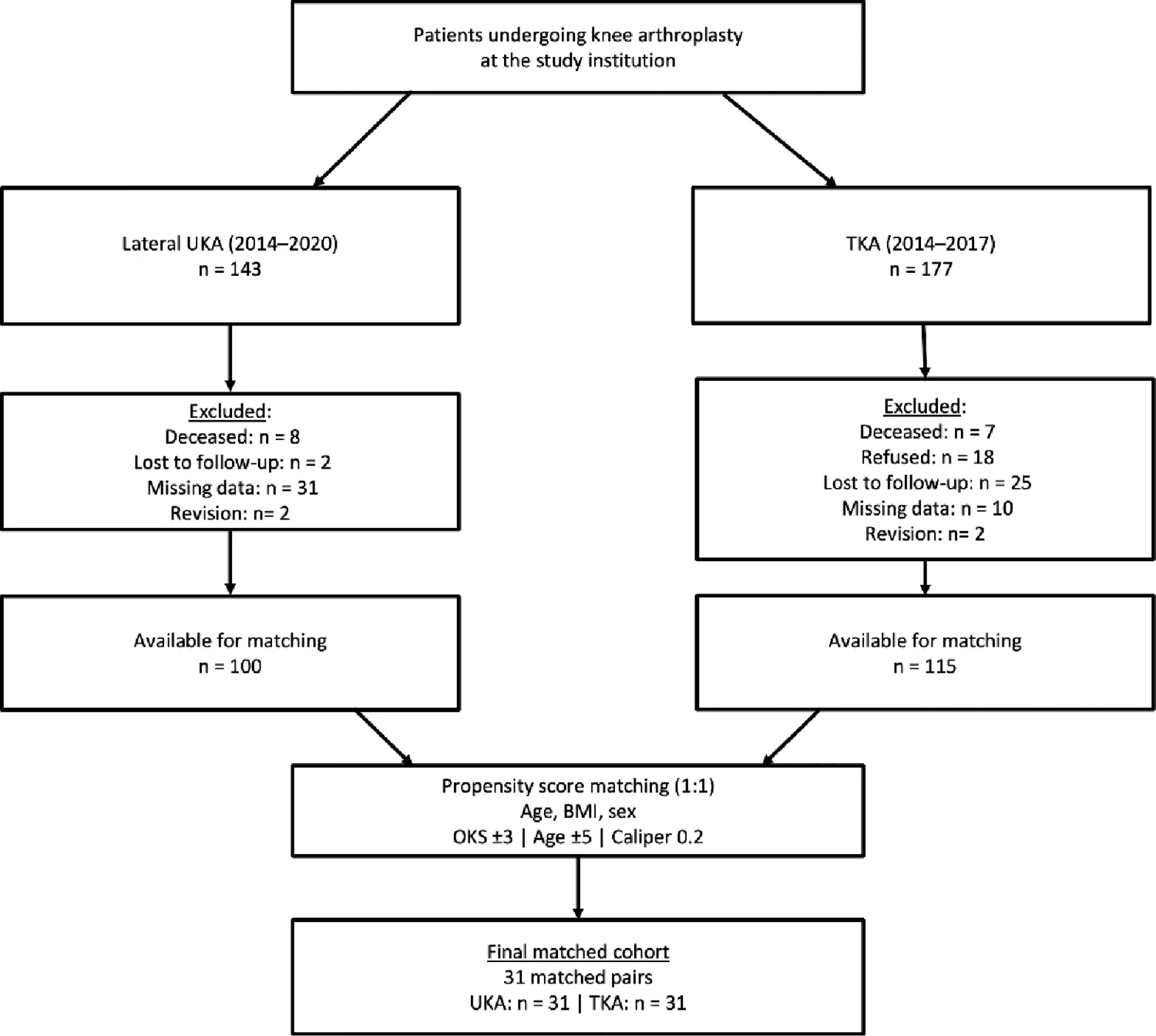
Flowchart illustrating patient selection, exclusions, and propensity score matching for the final study cohort comparing lateral unicompartmental knee arthroplasty and total knee arthroplasty. *BMI* body mass index; *OKS* Oxford Knee Score; *TKA* total knee arthroplasty; *UKA* unicompartmental knee arthroplasty

Matched cohort and baseline characteristics.

Of the 143 lateral UKA cases screened, 43 were excluded prior to matching because of death (*n* = 8), loss to follow-up (*n* = 2), missing postoperative PRO data (*n* = 31), or revision (*n* = 2) due to persistent pain in different hospitals, leaving 100 cases eligible for matching. Among 177 TKA procedures screened, 62 were excluded because of death (*n* = 7), refusal to participate (*n* = 18), loss to follow-up (*n* = 25), missing PRO data (*n* = 10), or revision (*n* = 2) resulting in 115 procedures eligible for matching (Fig. 1). In the TKA group, one revision was performed for suspected periprosthetic joint infection and consisted of debridement, antibiotics, and implant retention (DAIR) with polyethylene insert exchange, whereas the second revision was performed for symptomatic retropatellar osteoarthritis and consisted of secondary patellar resurfacing.

**Fig. 2:**
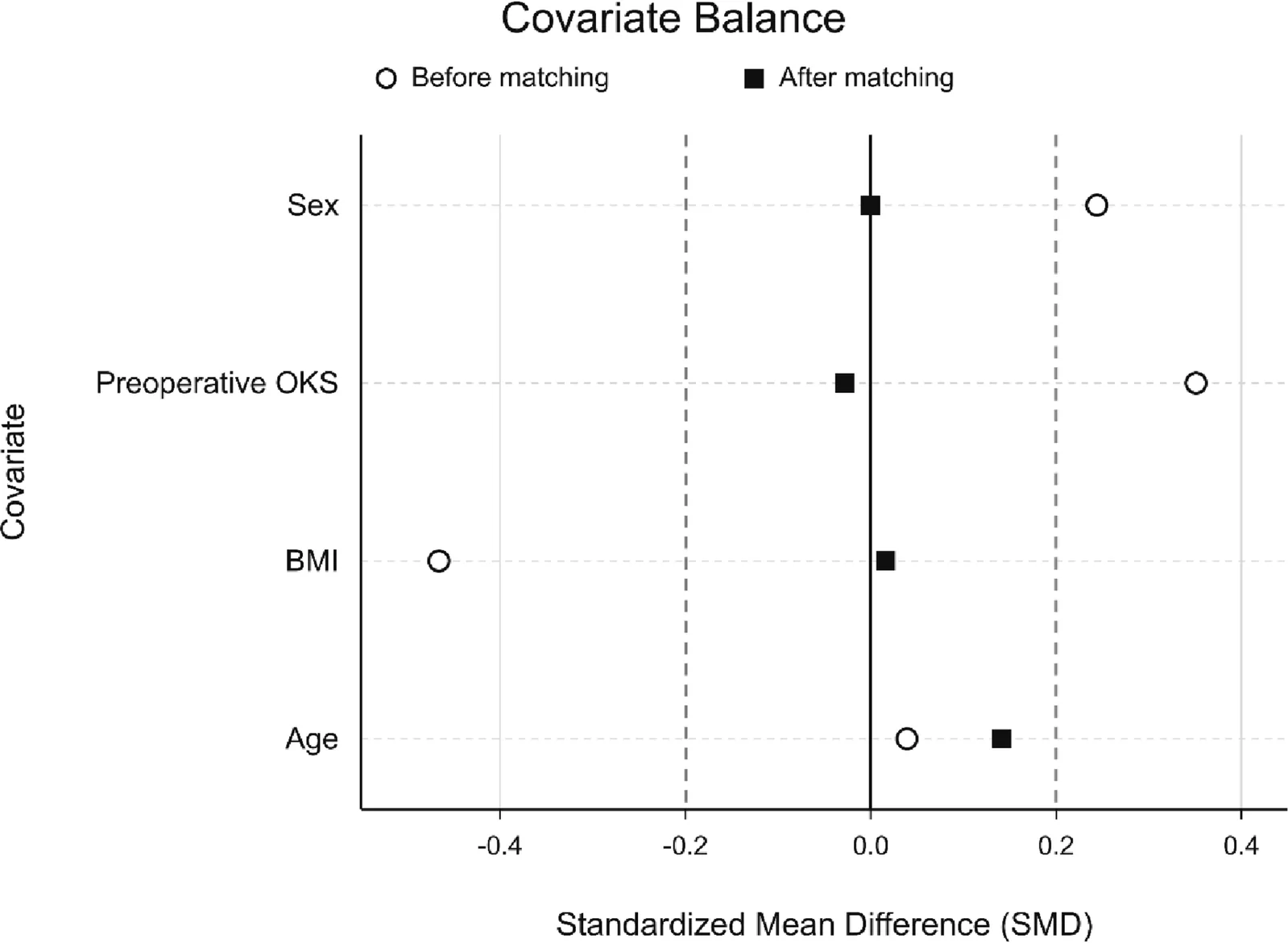
Standardized mean differences (SMDs) for baseline covariates before (circles) and after (squares) propensity score matching. Dashed vertical lines indicate thresholds of ± 0.20, which are commonly considered indicative of adequate balance. After matching, all covariates achieved sufficient balance within this range

PSM yielded 31 pairs. After matching, the absolute standardized mean differences were 0.141 for age, 0.016 for BMI, 0.028 for preoperative OKS, and 0.000 for sex. All measured variables were therefore below the predefined absolute SMD threshold of 0.20, although small residual differences remained for age (Fig. 2; Table 1).

Baseline characteristics are shown in Table 1. Mean follow-up was 4.1 ± 2.2 years (range: 1.0–8.5 years) after lateral UKA and 3.8 ± 1.1 years (range: 2.3–5.8 years) after TKA.

**Table 1:** Baseline characteristics of the study population before and after matching

Variable	Before matching		After matching				
	UKA (*n* = 100)	TKA (*n* = 115)	UKA (*n* = 31)	TKA (*n* = 31)	SMD		
Age at surgery (years)	64.1 ± 11.4	63.7 ± 9.1	67.2 ± 7.5	66.1 ± 7.1	0.141		
Female sex, *n* (%)	77 (77.0)	76 (66.1)	23 (74.2)	23 (74.2)	0.000		
BMI (kg/m²)	27.7 ± 5.3	30.7 ± 7.3	28.5 ± 4.6	28.4 ± 4.4	0.016		
Preoperative OKS	27.0 ± 7.9	24.3 ± 7.5	22.8 ± 5.8	23.0 ± 5.7	0.028		
Follow-up (years)	3.4 ± 1.8	3.9 ± 1.2	4.1 ± 2.2	3.8 ± 1.1	-		

Data are shown as mean ± standard deviation

*BMI* body mass index, *OKS* Oxford Knee Score, *TKA* total knee arthroplasty, *UKA* unicompartmental knee arthroplasty, *SMD* standard mean difference

### Clinical outcomes

**Fig. 3:**
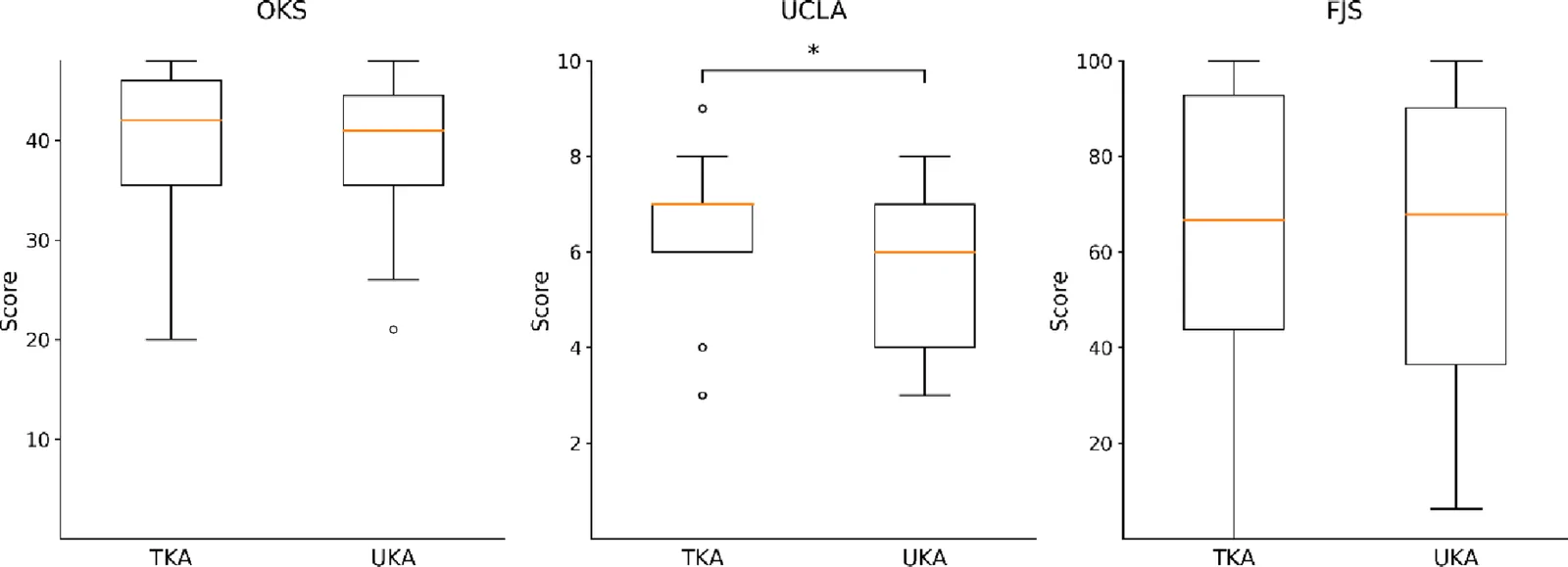
Boxplots illustrating postoperative outcomes after propensity score matching. Postoperative Oxford Knee Score (OKS; left), University of California, Los Angeles (UCLA) activity score (middle), and Forgotten Joint Score (FJS; right) are displayed for patients treated with total knee arthroplasty (TKA) and lateral unicompartmental knee arthroplasty (UKA). Boxes represent the interquartile range, with the median indicated by the horizontal line; whiskers extend to 1.5 times the interquartile range, and observations outside this range are displayed as individual data points. Between-group comparisons were performed using two-sided paired t-tests. The UCLA activity score differed significantly between groups (*p* = 0.008), whereas no statistically significant differences were detected for postoperative OKS or FJS. *p < 0.05

Postoperative outcomes are presented in Table 2; Fig. 3. Both groups showed substantial improvement in the OKS. The mean postoperative OKS did not differ significantly between UKA and TKA (*p* = 0.590). Similarly, no difference was found in the FJS (*p* = 0.900). In contrast, the UCLA activity score was significantly higher in the TKA group compared with the UKA group (*p* = 0.008).

The proportion of patients achieving the MCID in OKS was high in both groups (UKA: 90.3%, TKA: 93.5%), with no significant difference (exact McNemar *p* = 1.000). Similarly, the proportion of patients reaching the PASS for the FJS did not differ significantly between groups (UKA: 67.7%, TKA: 77.4%; exact McNemar *p* = 0.774).

**Table 2:** Patient-reported outcomes in the matched cohort

Outcome	UKA (*n* = 31)	TKA (*n* = 31)	Mean paired difference, TKA minus UKA (95% CI)	Cohen‘s d_z (95% CI)	*p*-value
OKS	38.9 ± 7.0	39.8 ± 7.5	0.94 (-2.57 to 4.44)	0.10 (-0.25 to 0.50)	0.590
∆OKS	16 ± 8.1	16.8 ± 7.4	0.77 (-2.62 to 4.17)	0.08 (-0.27 to 0.47)	0.644
UCLA-Score	5.5 ± 1.5	6.3 ± 1.7	0.93 (0.26 to 1.60)	0.53 (0.18 to 0.95)	0.008 *
FJS	64.0 ± 29.6	64.1 ± 28.8	0.83 (-12.53 to 14.19)	0.02 (-0.33 to 0.43)	0.900

* *p* < 0.05

Continuous outcomes are presented as mean ± standard deviation. Mean paired differences were calculated as TKA minus UKA; positive values indicate higher scores after TKA.

*CI* confidence interval, *FJS* Forgotten Joint Score, *MCID* minimal clinically important difference, *OKS* Oxford Knee Score, *PASS* patient acceptable symptom state, *UCLA* University of California, Los Angeles activity score.

## Discussion

The primary finding was that no statistically significant between-group difference was detected in postoperative OKS. No statistically significant differences were detected in pre-to-postoperative OKS improvement, FJS, OKS MCID achievement, or FJS PASS attainment, whereas postoperative UCLA activity scores were higher in the TKA group.

These results are consistent with previous comparative studies demonstrating that the functional advantages often attributed to UKA over TKA diminish when baseline differences are adequately controlled [19, 23]. Several analyses have shown that superior postoperative scores after UKA are frequently driven by higher preoperative function, younger age, and more favorable disease patterns rather than by the procedure itself. When patients are matched for key demographic and functional variables, differences in PROs between UKA and TKA tend to be small and often fall below established thresholds for clinical relevance [22, 26, 37]. While a large propensity-matched analysis of over 38,000 UKA and TKA cases found that UKA had higher mean postoperative OKS and quality-of-life scores, the difference was below the MCID [26]. Similarly, a meta-analysis of randomized controlled trials found that although UKA was associated with statistically better knee function and less pain, these differences did not reach the MCID, indicating no clear clinical advantage when selection effects are accounted for [37]. However, both the registry analysis by Mohammad et al. and the meta-analysis by Xia et al. evaluated UKA in general, without stratification by compartment, and therefore predominantly represent medial UKA given the epidemiologic distribution of knee OA. Our findings provide additional lateral UKA-specific data within this context.

The mean OKS and FJS of both groups in this study population are comparable to earlier reported results of patients following lateral UKA [2, 17], but higher than those after TKA in previous studies [5, 10, 31]. This finding may partly reflect the PSM strategy applied in the present analysis. By matching TKA patients to lateral UKA recipients on age, sex, BMI, and preoperative OKS, the analysis selected a TKA subgroup whose measured baseline profile more closely resembled that of the lateral UKA cohort than that of an unselected TKA population. This selected TKA subgroup may therefore differ from typical unselected TKA cohorts in characteristics associated with postoperative function, potentially contributing to the comparatively high postoperative OKS values observed in the present study.

Although matched and randomized comparisons between UKA and TKA are available, most previous studies have focused on medial UKA or have combined medial and lateral procedures. More recently, Lu et al. compared lateral UKA and TKA in 115 patients with isolated lateral-compartment OA and anterior knee pain treated within a standardized lateral parapatellar pathway. They reported faster early recovery and a lower perioperative burden after lateral UKA, whereas no statistically significant between-group differences were detected in KSS, WOMAC, or compartment-specific pain at 24 months [21]. Similarly, no statistically significant between-group differences in postoperative OKS or FJS were detected in our cohort. However, differences in study population, surgical approach, follow-up interval, and outcome instruments preclude direct comparison. Importantly, unlike the study by Lu et al., our TKA cohort predominantly consisted of patients with multicompartmental OA, and the two procedures therefore did not represent treatment alternatives within the same indication [21]. The present study provides complementary evidence by examining postoperative OKS, pre-to-post OKS change, FJS, UCLA activity scores, OKS MCID achievement, and FJS PASS attainment in propensity score-matched cohorts.

The activity level of the study population measured by the UCLA score was high and comparable to values in previously published studies on lateral UKA and TKA [9, 13, 28, 38]. While Meena et al. reported no significant difference in sports participation or activity levels between UKA and TKA after controlling for baseline variables [23], the current study demonstrated higher UCLA scores for the TKA group. However, because preoperative UCLA scores were unavailable, it remains unclear whether this difference reflects a treatment-related effect or pre-existing differences in activity level.

Lateral UKA nevertheless continues to offer clinically relevant advantages over TKA, particularly in younger and more active patients [15, 35]. The less invasive nature of UKA, preservation of bone stock, and retention of cruciate ligaments, contribute to a more physiological joint kinematic profile, which is associated with faster recovery, lower perioperative morbidity, reduced blood loss, and shorter hospital stays [16, 20, 32]. These benefits are especially relevant in younger patients, in whom faster return to activity and reduced surgical morbidity are highly valued and may influence treatment preference.

In addition to the extent and biomechanics of joint replacement, the procedures differed in surgical approach: lateral UKA was performed through a lateral parapatellar approach, whereas TKA was performed through a medial parapatellar approach. These differences may influence component positioning, patellofemoral mechanics, and patient-perceived recovery. In a retrospective comparison of two approaches for lateral UKA, Keppler et al. found more frequent deviations in femoral component flexion and more frequent lateral positioning of the femoral component with the transpatellar lateral approach than with a Hoffa’s fat pad–preserving medial approach, indicating that surgical exposure may affect the accuracy of component positioning [18]. Component position may, in turn, influence patellofemoral symptoms. Hernigou and Deschamps reported that patellar impingement occurred more frequently after lateral than medial UKA and was associated with excessively anterior placement of the femoral component [14]. Patellofemoral complications were associated with more frequent pain during stair climbing and rising from a chair, with symptoms being more severe in knees with patellar impingement. By contrast, D’Ambrosi et al. found no significant pre- to postoperative or between-group differences in the Insall–Salvati or Caton–Deschamps indices after medial versus lateral UKA, suggesting no systematic difference in radiographic patellar height [6]. Nevertheless, these studies provide only indirect mechanistic explanations and do not establish that the surgical approach caused the differences in PROs observed in our cohort. Because surgical approach, implant design, ligament preservation, and underlying indication were intrinsically linked to the type of arthroplasty performed, their independent effects could not be determined.

Several limitations of this study should be acknowledged. First, the retrospective design limits causal inference and is inherently susceptible to selection bias.

The small number of matched pairs limits the statistical precision of the estimates and increases the possibility of type II error. Therefore, the absence of statistically significant differences does not exclude potentially clinically relevant between-group differences.

Most importantly, indication bias represents a key limitation: in our institution, lateral UKA was implanted whenever feasible, whereas TKA was predominantly used in patients with more advanced or multicompartmental OA. Although PSM was applied to balance demographic variables, residual confounding related to disease distribution, cartilage status of non-replaced compartments, and ligament integrity cannot be fully excluded.

The matched TKA cohort may therefore represent a selected subgroup with relatively preserved preoperative function despite more extensive structural disease. Conversely, selection of UKA cases with lower preoperative function may have attenuated a potential UKA advantage. These selection mechanisms may bias the between-group estimates in different directions, and their net effect cannot be quantified. Accordingly, the findings are descriptive and cannot establish causal comparative effectiveness or equivalence in patients eligible for both procedures. The selection of the appropriate surgical procedure should remain individualized according to disease pattern, patient characteristics and expectations, and surgeon expertise.

Missing postoperative outcome data affected 31 of 143 UKA cases (21.7%) and 10 of 177 TKA procedures (5.6%). In addition, 2 UKA cases and 25 TKA procedures were lost to follow-up. Because the probability of outcome availability may have been related to postoperative function and satisfaction, the assumption that these data were missing completely at random cannot be made. Differential missingness between the cohorts may therefore have introduced attrition bias. In addition, because the latest available assessment rather than a uniform postoperative time point was analyzed, follow-up duration was heterogeneous. Although mean follow-up durations were similar, the wider follow-up range in the UKA group may have introduced time-related heterogeneity in the PROs. Furthermore, preoperative UCLA scores were unavailable, precluding adjustment for baseline activity and limiting the interpretation of the statistically significant postoperative UCLA difference.

At the same time, a major strength of this study lies in the rigorous matching strategy, which included not only demographic factors but also preoperative functional status. Matching on baseline function is particularly important in comparative arthroplasty research, as preoperative PROs strongly influence postoperative outcomes. By reducing imbalance in demographic characteristics and preoperative OKS, the present analysis provides a more balanced assessment of procedure-related differences than unmatched or registry-based comparisons.

## Conclusion

The findings of this study suggest that the theoretical advantages of joint preservation do not necessarily translate into superior PROs once demographic characteristics and preoperative function are balanced. Nevertheless, the substantial differences in surgical indications preclude conclusions regarding comparative effectiveness. Procedure selection should therefore remain indication-based: lateral UKA represents a joint-preserving option for carefully selected patients with isolated lateral-compartment OA, whereas TKA provides reliable PROs in patients requiring more extensive joint replacement.

## Data Availability

The data are available from the corresponding author upon reasonable request.

## Abbreviations

BMI: Body mass index
CI: Confidence interval
CR: Cruciate-retaining
FJS: Forgotten joint score
MCID: Minimal clinically important difference
OA: Osteoarthritis
OKS: Oxford knee score
PASS: Patient acceptable symptom state
PROs: Patient-reported outcomes
PS: Posterior-stabilized
PSM: Propensity score matching
SD: Standard deviation
SMD: Standardized mean difference
TKA: Total knee arthroplasty
UCLA: University of California Los Angeles activity scale
UKA: Unicompartmental knee arthroplasty
